# Editorial: Impaired oxygen delivery in experimental disease models: Pathogenesis, diagnostics and treatment strategies

**DOI:** 10.3389/fmed.2022.995958

**Published:** 2022-08-25

**Authors:** Wolfgang Weihs, Andrey V. Kozlov, Hiroshi Saito, Johanna Catharina Duvigneau

**Affiliations:** ^1^Department of Emergency Medicine, Medical University of Vienna, Vienna, Austria; ^2^Ludwig Boltzmann Institute for Traumatology, The Research Center in Cooperation With Allgemeine Unfallversicherungsanstalt (AUVA), Vienna, Austria; ^3^Austrian Cluster for Tissue Regeneration, Vienna, Austria; ^4^Department of Surgery, University of Kentucky, Lexington, KY, United States; ^5^Department of Biomedical Sciences, University of Veterinary Medicine, Vienna, Austria

**Keywords:** oxygen delivery, hemorrhagic shock, systemic inflammatory response, ischemia reperfusion injury, metabolism, hypoxia, hypothermia

This Research Topic is aimed at collecting contributions that help to shed more light on pathogenesis, diagnostics, and prospective therapeutic tools in critical clinical conditions resulting from impaired oxygen availability, such as cardiac arrest, hypoxia, ischemia, and sepsis. Emphasizing translational research, we invited the scientific community to contribute studies conducted with experimental animal models, as these allow the detailed analysis of the complex pathophysiological processes for developing new therapeutic strategies. Here we present nine contributions. Seven studies describe experimental results, mainly animal experiments, and two are reviews that expand our knowledge of the physiological and pathophysiological events associated with impaired oxygen delivery.

The review of Hof, Marcus et al. offers a “tool-box,” comprising a combination of analyses to determine structural and functional changes of the microcirculation and of mitochondria that are applicable in studies using experimental animals for modeling septic complications and hemorrhagic shock. In order to accurately assess the consequences of impaired oxygen delivery on organ function, cell metabolism, and inflammatory processes, the authors further suggest magnetic resonance imaging as a non-invasive method.

The review of Bjertnæs et al. gives us a well-written overview about the physiological consequences of accidental hypothermia, a medical condition, which still has a high mortality rate. The authors describe guidelines for the rewarming procedure, which are of relevance not only for the treatment of victims of accidental hypothermia, but may also interest those considering hypothermia as a therapeutic approach.

An additional insight in the association of hypothermia and hypoxia is given in the *in-vitro* study presented by Woyke et al., describing the combined effect of lowering temperature and increasing CO_2_ levels on the oxygen dissociation curve in unbuffered human whole blood. The authors found that decreasing temperature leads to an increased oxygen affinity to hemoglobin. However, this temperature effect is outweighed by the CO_2_-effect, inducing a right shift of the oxygen dissociation curve. Interestingly, the relative CO_2_-effect was higher with decreasing temperature, indicating a significant interaction of temperature and pCO_2_. Additionally, the CO_2_-effect was diminished at higher CO_2_ levels suggesting saturation effect. The study further suggests that levels of pCO_2_ exceeding 40 mmHg may result in a too poor oxygen binding and an insufficient oxygen delivery within the system.

This study presented by Hof, Truse et al. compared the effect of locally applied CO_2_ and O_2_ on gastric and oral microcirculation in dogs subjected to hemorrhagic shock. The idea behind it was, that when applied locally, CO_2_ at high concentrations increases the oxygen release from hemoglobin locally, which can be used to intentionally improve the oxygenation of hypoxic tissue. Indeed, local hypercapnia improved microvascular oxygenation and was associated with a continuous blood flow. Thus, local CO_2_-application is able to improve oxygen saturation of hypoxic tissues better than application of oxygen and represents an interesting new minimally invasive approach to improve gastric microcirculation during hemorrhagic shock.

A deeper insight into the cellular metabolism associated with insufficient oxygen supply is given by the contribution of Graf et al. They used succinyl phosphonate as an inhibitor of 2-oxoglutarate dehydrogenase complex (OGDHC) in cerebellar tissue of pregnant and non-pregnant rats. Both, OGDHC inhibition by limiting NADH, and hypobaric hypoxia by limiting oxygen supply, perturb the respiratory chain function. OGDHC inhibition produced similar changes in the cerebellar amino acid pools, which were different between pregnant and non-pregnant rats. The authors suggest that chemical OGDHC inhibition is suitable for mimicking the metabolic changes induced by insufficient oxygen supply.

The study by Rutai et al. used a clinically relevant porcine model for mimicking human sepsis and septic shock and developed a standardized research protocol that allowed characterizing the progression of sepsis-related events. Their approach is to cluster the host responses into sepsis and septic shock groups using a specific porcine SOFA score including quantitative and qualitative assessment of blood CFUs along with the measurement of macro- and microcirculatory variables.

The study by Denoix et al. used a porcine long-term model of hemorrhagic shock and resuscitation with pre-existing atherosclerosis to investigate whether sodium thiosulfate, an H_2_S releasing compound, exerts neuroprotective effects. However, despite its lung-protective efficacy in this model, neuro-histopathological analysis revealed no differences between groups, possibly because the blood brain barrier remained intact and neuronal tissue appeared relatively unaffected by the induced hemorrhagic shock. This research group additionally examined H_2_S as a potential therapeutic in mice with deleted H_2_S-producing cystathionine-γ-lyase (CSE). In a previous study, they could demonstrate sodium thiosulfate-mediated protection against traumatic/hemorrhagic shock induced injury (1).

Using the same model, the group next investigated the role of sodium thiosulfate in the CSE knock-out mice challenged with an underlying co-morbidity, such as diabetes. The data, which are presented by Gröger et al., show that traumatic/hemorrhagic shock in streptozotocin-induced diabetic mice led to severe circulatory failure, strong manifestation of an inflammatory response, and an increased tissue expression of typical stress response markers, however, none was prevented by the application of sodium thiosulfate. These findings are of translational significance, since they show that an underlying co-morbidity not only worsens the shock induced pathophysiological changes, but may also diminish efficacy of the therapeutic approach.

Finally, the contribution of Szabó-Biczók et al. dealt with the side effects of therapeutic restoration of oxygen supply using veno-venous extracorporeal membrane oxygenation (ECMO). Although ECMO can save lives in respiratory distress, it has serious side-effects as it frequently induces acute kidney injury. Therefore, the group developed a clinically relevant model using Vietnamese minipigs for a prolonged ECMO protocol (30 h). The described protocol induced acute renal impairment, which was demonstrated by a significantly decreased renal function with signs of structural damage and impaired mitochondrial function. It is therefore a good tool to further study therapeutic interventions for decreasing acute kidney injury introduced by ECMO.

## Author contributions

JCD and WW planned the Editorial. WW wrote the first draft. JCD and AVK performed major revisions of the text. All authors contributed and approved the submitted version of the article.

## Conflict of interest

The authors declare that the research was conducted in the absence of any commercial or financial relationships that could be construed as a potential conflict of interest.

## Publisher's note

All claims expressed in this article are solely those of the authors and do not necessarily represent those of their affiliated organizations, or those of the publisher, the editors and the reviewers. Any product that may be evaluated in this article, or claim that may be made by its manufacturer, is not guaranteed or endorsed by the publisher.
